# Obesity alters composition and diversity of the oral microbiota in patients with type 2 diabetes mellitus independently of glycemic control

**DOI:** 10.1371/journal.pone.0204724

**Published:** 2018-10-01

**Authors:** Jonathan Tam, Thomas Hoffmann, Sabine Fischer, Stefan Bornstein, Jürgen Gräßler, Barbara Noack

**Affiliations:** 1 Department of Periodontology, Medical Faculty Carl Gustav Carus, TU Dresden, Dresden, Germany; 2 Department and Outpatient Department of Medicine III, University Hospital Carl Gustav Carus, TU Dresden, Dresden, Germany; University of the Pacific, UNITED STATES

## Abstract

**Background and objective:**

The involvement of the oral microbiota as a possible link between periodontitis, type 2 diabetes mellitus and obesity is still not well understood. The objective of the study was to investigate if glycemic control and obesity play a role in modulating the composition and diversity of the oral microbial ecology.

**Material and methods:**

A cohort of patients with type 2 diabetes mellitus (n = 18) was recruited. Participants demonstrating improved glycemic control after 3 months (n = 6) were included in a second examination. A full mouth examination was performed to estimate periodontitis severity followed by sample collection (subgingival plaque and saliva). Generation of large sequence libraries was performed using the high-throughput Illumina MiSeq sequencing platform.

**Results:**

The majority of participants (94.4%, n = 17) presented with moderate or severe forms of periodontitis. Differences in microbial composition and diversity between obese (BMI ≥ 30 kg/m^2^) and non-obese (BMI < 30 kg/m^2^) groups were statistically significant. Cross-sectional and longitudinal approaches failed to reveal statistically significant associations between HbA_1c_ level and species composition or diversity.

**Conclusions:**

Obesity was significantly associated with the oral microbial composition. The impact of glycemic control on oral microbiota, however, could not be assured statistically.

## Introduction

There is mounting evidence that the intestinal microbiota may have an impact on both type 2 diabetes mellitus and obesity through alterations of metabolic processes in glucose and fatty acid metabolism pathways [1–4]. This impact is not only attributed to the composition of the intestinal microbiota, but also to a change in the bacterial diversity within the gut [3,5,6]. Interventional studies performed in the human and mouse model using fecal microbiota transplantation have shown that the gut microbiota plays a key role in weight regulation as well as in insulin sensitivity [7–9].

Periodontitis is the result of bacterially induced inflammation that extends into gum tissue leading to gradual destruction of connective tissues and alveolar bone [10]. Furthermore, periodontal inflammation has been linked to various systemic diseases with high prevalence, incidence, morbidity, and mortality; for example type 2 diabetes mellitus [11] and obesity [12]. In both cases, the pro-inflammatory milieu associated with both obesity and type 2 diabetes mellitus may play a key role in the mechanisms linking these two diseases with periodontal disease [13,14]. However, Shungin et al. argued that observational studies are sensitive to confounding, bias and reverse causality, and by employing Mendelian randomization causal analyses, concluded that total adiposity is unlikely to be causally related with periodontitis [15]. On the other hand, a 'two-way' interaction between type 2 diabetes mellitus and periodontitis is well-established [14], and implicates that not only is diabetes a risk factor for periodontitis, but periodontitis could have a negative effect on glycemic control.

Studies of the human microbiome and obesity have mainly focused on the distal gut and fecal microbiome samples, with less attention paid to the microbial composition in the upper gastrointestinal tract. Tsuda et al. [16] have demonstrated a high level of similarity, in both diversity and in composition, between the microbiotas of the oral cavity and the upper gastrointestinal tract. Interestingly, the fecal microbiota was shown to greatly differ from those of saliva and gastric fluid. Studies in mice have shown that there exists a causative link between oral pathogens and changes in the gut microbiota and in inflammatory status [17,18]. Hence, the aim of this study was to test the hypothesis that the composition and diversity of the oral microbiota may be associated with the glycemic state and body weight.

## Methods

### Study design and participants

This mono-centric, prospective cohort study was conducted at the University Hospital Dresden in the Department of Periodontology and in the Department and Outpatient Department of Medicine III. The target population included patients with poorly controlled type 2 diabetes mellitus in ambulant treatment with need of glycosylated haemoglobin (HbA_1c_) improvement. The inclusion criteria were: 18 to 80 years of age, type 2 diabetes mellitus as diagnosed following the recommendations of the American Diabetes Association (HbA_1c_ ≥ 6.5%) [19], and at least 10 teeth and/or implants, wisdom teeth excluded. Patients were not included in the study when the following diseases and conditions were present: type 1 diabetes, endocarditis prophylaxis required, pregnancy or breastfeeding, incapability of assessing essence and possible consequences of the study, lack of compliance, consumption of more than five cigarettes per week, alcohol consumption exceeding 40 g/alcohol per day, requirement for medications known to influence the gingival condition (e.g. phenytoin, nifedipine, immune suppressive therapy, steroids, antiphlogistics), treatment with antibiotics three months prior to start of study, myocardial infarction and/or stroke within 2 years of start of study, current treatment of tumor disease, macroalbuminuria exceeding 300 mg/L albumin in urine and/or dialysis dependency, severe liver disorders (gamma-glutamyltransferase and alanin-aminotransferase activities exceeding 2 μmol/L x s), current participation in another study.

A total of 69 patients were questioned for participation. 51 patients either declined to participate or did not satisfy the necessary criteria. 18 participants were recruited for the study. Patients were motivated to exercise regularly and a healthy diet was promoted. Furthermore, oral antidiabetic medication and/or insulin were optimized. A significant improvement of glycemic control (IGC) was defined by an HbA_1c_ reduction of at least 0.5% during the observation period of 3 months. Six participants demonstrated IGC and a follow up examination was performed 3 months after baseline. Comparisons were also made between obese and non-obese patients. Obesity was defined by body mass index (BMI) ≥ 30 kg/m^2^. Fasting blood samples were collected in the morning and processed within the same day. Fasting was defined as no caloric intake for ≥ 8 h. Laboratory parameters included fasting plasma glucose, HbA_1c_, triglyceride, total cholesterol, and LDL- and HDL-cholesterol concentrations.

All study participants received an appropriate description of the study protocol. Written informed consent was obtained before the study was performed, and the study protocol was approved by the Ethics Committee of the Technische Universitaet Dresden (EK 42022014) in accordance with the Declaration of Helsinki.

### Oral examination and oral sample collection

Oral examination and sample collection was performed between 8:00 AM and 2:00 PM. All patients were asked to refrain from eating or tooth brushing for 1 h before oral examination and sample collection.

Participants underwent a complete periodontal examination. One trained and calibrated examiner (B.N.) conducted all periodontal measurements. Data collected included: periodontal probing depth (PD), clinical attachment loss (CAL) and bleeding on probing (BOP), all at six sites per tooth. A modification of the Silness-Löe plaque index (PI) was used to record plaque accumulation to determine oral hygiene status [20]. The diagnosis of periodontitis was assigned following recent consideration of diagnostic criteria for periodontal diseases [21,22]. Immediately after the oral examination, subgingival samples were collected at the deepest PD from each quadrant using sterile universal curettes after removal of supragingival plaque with a sterile swab and pooled together. Samples were transferred into sterilized microfuge tubes. Unstimulated whole saliva was collected by passive drooling into sterile plastic tubes and transferred into sterile microfuge tubes after collection of approximately 2 mL of whole saliva. All samples were immediately frozen at - 80°C.

### DNA extraction and MiSeq sequencing

Bacterial chromosomal DNA extraction and purification was performed using the QIAamp DNA Stool Kit (Qiagen, Valencia, CA, USA). The DNA-isolation for Pathogen Detection Protocol was performed in accordance with the manufacturer’s guidelines with two modifications: (i) 0.7 mL of the ASL buffer was added to each sample instead of the recommended 1.4mL, and (ii) InhibitEX tablets were previously halved and added to each sample. DNA concentration and sample purity were estimated by Nanodrop (Thermo Scientific, San Diego, CA, USA). Bacterial 16S rRNA gene amplification, sequencing of the polymerase chain reaction products and subsequent diversity analysis were performed blinded at BGI Genomics Co, Ltd (Shenzhen, China). A region of approximately 469 base pairs encompassing the V3 and V4 hypervariable regions of the 16S rRNA gene was amplified using polymerase chain reaction and modified primers (341F: 5’-ACTCCTACGGGAGGCAGCAG-3’), 806R: 5’-GGACTACHVGGGTWTCTAAT-3’) [23]. The PCR conditions were as follows: 2 minutes of initial denaturation at 95°C followed by 30 cycles of denaturation at 95°C (20 s), annealing at 56°C (30 s), elongation at 72°C (45 s) and a final extension at 72°C for 7 minutes. Following purification of the amplicon pools using AMPure XT beads, sequencing was consequently performed on the Illumina MiSeq platform (San Diego, CA, USA) using the 300 PE MiSeq run.

### Microbiome analyses

Overlapping paired-end reads were used to generate consensus sequences using Fast Length Adjustment of Short Reads (version 1.2.11) [24]. Clustering of tags into operational taxonomic units (OTUs) with a 97% threshold was obtained using UPARSE [25]. Chimeras were removed using UCHIME v.4.2.40 [26]. Taxonomic annotation was performed using the Ribosomal Database Project Classifier (version 2.2) [27] trained on the GreenGenes database (version 201305) [28] as well as the Human Oral Microbiome Database [29].

### Statistical analysis

Statistical analyses were performed with the SPSS software version v22 (Statistical Package for the Social Sciences; SPSS, Chicago, IL). Alpha and beta diversity metrics were calculated to analyse within and between sample complexities. The non-parametric Wilcoxon-rank-sum test was utilized for comparing different sample groups as well as for comparisons between baseline and follow up examination. Correlation testing between diversity metrics and glycemic control or obesity was performed using Spearman's correlation analysis. The linear discriminant analysis (LDA) effect size (LEfSe) algorithm for high dimensional metagenomic biomarker discovery was used for identifying differentially abundant features between different conditions [30]. All analyses were run with LEfSe's α parameter for pairwise tests set to 0.05 and the threshold of the logarithmic score for LDA analysis set to 2.0.

Stringent multiple test correction was applied using the Benjamini Hochberg false discovery rate (FDR) [31]. Multidimensional scaling (MDS) was used for visualizing the level of similarity between species complexity before and after improved glycemic control.

Sample size calculation was performed based on a correlation analysis of glycemic control (HbA1c) with alpha and beta diversity parameters of OMB. The software package G*Power version 3.19.2, University Duesseldorf, Germany was used [32]. Assuming a moderate correlation, N = 16 plaque or saliva samples were necessary to detect a significant correlation between HbA1c and diversity indices (power = 0.8 and α = 0.05) [33].

## Results

### General findings

Characteristics of all study patients are summarized in Table 1. All of the participants in the study (n = 18) were prescribed oral antidiabetic medication and/or insulin. Of the 18 examined participants, 15 underwent statin therapy for management of blood cholesterol during the examination period. The majority of participants (94.4%, n = 17) presented with moderate or severe forms of periodontitis. The correlation between HbA_1c_ and BMI was significant (r _Spearman_ = 0.49, p = 0.039).

**Table 1:** Baseline demographic, periodontal, and metabolic data of the study population (mean ±SD) or number (%) of participants, n = 18.

Male/female (number, %)	10 (55.6)/ 8 (44.4)
Age (years)	68.17 ± 6.83
BMI (kg/m^2^)	31.08 ± 6.09
BMI ≥ 30 kg/m^2^ (number, %)	6 (33.3)
Waist-hip ratio	0.96 ± 0.06
Oral antidiabetic medication (number, %)	7 (38.9)
Insulin (number, %)	3 (16.7)
Oral antidiabetic medication and Insulin (number, %)	8 (44.4)
HbA_1c_ (%) (minimum—maximum)	8.22 ± 1.44 (6.8–13.5)
Total cholesterol (mmol/l)	5.08 ± 2.23
Low-density lipoprotein cholesterol (mmol/l)	2.74 ± 0.97
High-.density lipoprotein cholesterol (mmol/l)	1.24 ± 0.31
Triglycerides (mmol/l)	3.14 ± 5.45
Number of teeth (n)	21 ± 5
PI	1.40 ± 0.61
BOP (% sites)	30.25 ± 19.82
CAL (mm)	3.39 ± 0.77
PD (mm)	2.67 ± 0.47
CAL proximal ≥ 4 mm (% sites)	34.78 ± 25.63
CAL proximal ≥ 6 mm (% sites)	6.25 ± 8.85
PD ≥ 4 mm (% sites)	15.89 ± 14.20
PD ≥ 6 mm (% sites)	1.44 ± 2.56
Periodontitis n (%)	
Moderate periodontitis^a^	7 (38.9%)
Severe periodontitis ^a^	10 (55.6%)
Generalized periodontitis ^b^	8 (44.4%)

^a^ CDC/AAP periodontitis case definition [22]

^b^ Generalized periodontitis definition [21]

An average of 28139 valid tags per sample was obtained from 48 samples: saliva and pooled subgingival plaque samples at baseline n = 18 each, saliva and pooled subgingival plaque after IGC n = 6 each. In general, more valid tags were collected in saliva samples (28892 tags on average) than in plaque samples (27385 tags on average). Valid tags were clustered into OTUs at 97% similarity. A total of 386 distinct OTUs were identified in plaque and saliva samples. Of which, 358 were shared between both sample types; and 8 and 20 OTUs were exclusive to plaque and saliva respectively. Estimations of species richness and diversity in plaque and saliva by calculating alpha diversity metrics reported a statistically significant different distribution of species richness between plaque and saliva at baseline. On average, more OTUs were observed in saliva samples (Table 2). A difference in species diversity between plaque and saliva was found at least in trend (estimated by Shannon index and Simpson index, Table 2). Abundance values of all identified OTUs are presented in the supplemental material (S1 Table).

**Table 2:** Mean alpha diversities of plaque and saliva samples at baseline as calculated using different alpha diversity metrics (n = 18).

		Plaque	Saliva	P-value ^a^
**Observed OTUs**	Mean ± SD	165.72 ± 35.51	182.61 ± 46.27	0.025
	Median (IQR)	165.00 (142.50; 195.00)	194.50 (142.50; 222.00)	
**Chao1**	Mean ± SD	186.07 ± 38.01	202.02 ± 48.64	0.022
	Median (IQR)	180.02 (168.92; 213.80)	208.57 (157.89; 243.54)	
**Shannon**	Mean ± SD	3.19 ± 0.62	3.41 ± 0.42	0.078
	Median (IQR)	3.38 (2.58; 3.71)	3.40 (3.16; 3.72)	
**Simpson**	Mean ± SD	0.11 ± 0.08	0.07 ± 0.03	0.022
	Median (IQR)	0.09 (0.05; 0.19)	0.07 (0.05; 0.09)	

IQR, Interquartile range

^a^ Wilcoxon test

**Fig 1:**
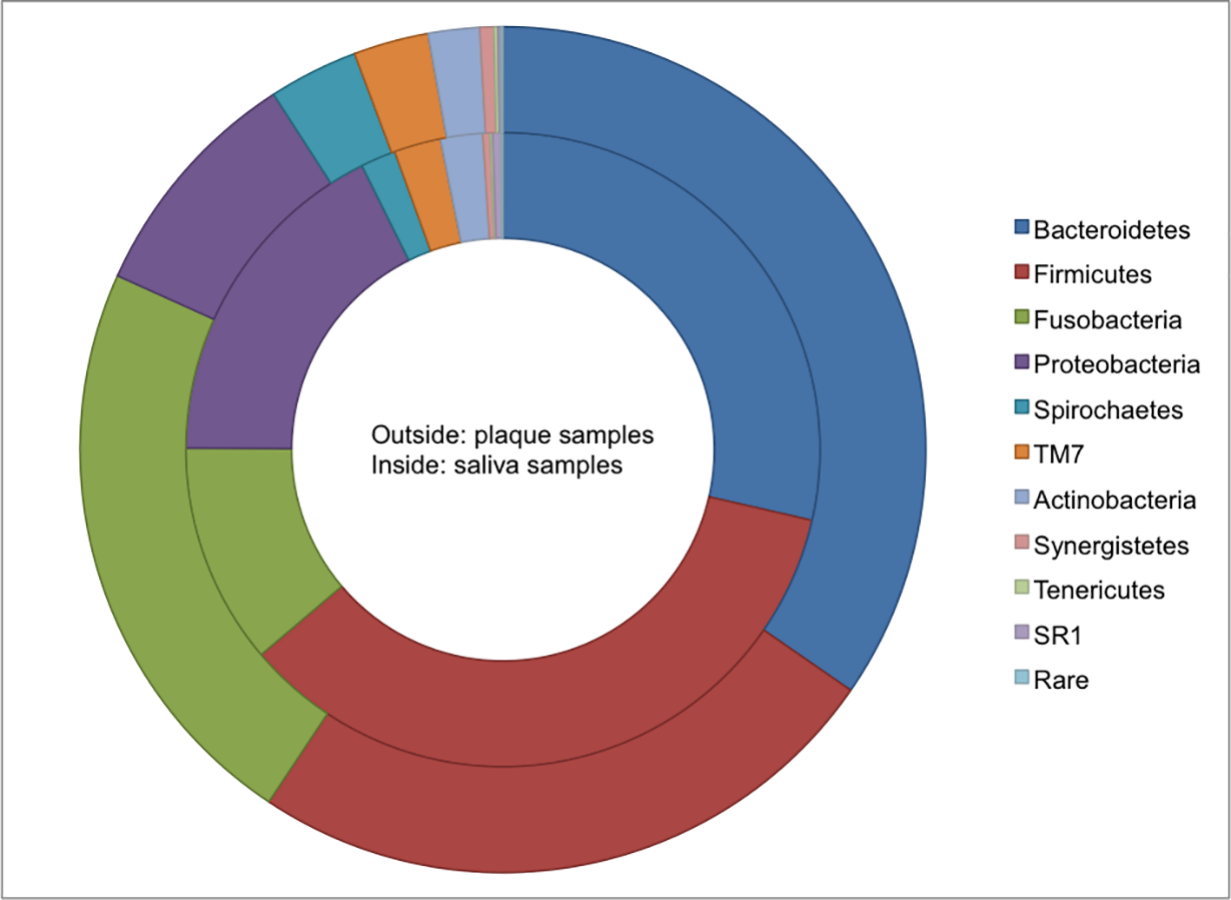
Comparison of the average taxonomy composition distribution at the phylum level in all plaque (outside) and saliva (inside) samples. *GN02*, *Elusimicrobia*, *Chloroflexi* and *Cyanobacteria* have been classified as 'Other' due to low abundance levels <0.1%.

A total of 14 phyla was detected (Fig 1). In descending order, the six most dominant phyla present in plaque were *Bacteroidetes* (34.63%), *Firmicutes* (24.71%), *Fusobacteria* (22.40%), *Proteobacteria* (9.13%), *Spirochaetes* (3.42%) and *TM7* (2.87%). Saliva samples, on the other hand, displayed different dominant phyla: (in descending order) *Firmicutes* (35.23%), *Bacteroidetes* (28.58%), *Proteobacteria* (17.59%), *Fusobacteria* (11.27%), *TM7* (2.39%) and *Spirochaetes* (1.78%). Despite distinct hierarchal distributions of taxa in plaque and in saliva, the six most dominant phyla in this study population comprised approximately 96% of total detected phyla.

### Glycemic status and composition of oral microbiome

No statistically significant associations between glycemic level and species composition or species diversity were found. Univariate Spearman's correlation analysis of the entire study population revealed no statistically significant correlation between HbA_1c_ and community richness or evenness as measured by different alpha diversity indices (Table 3). While few bacterial taxa initially demonstrated a correlation with HbA_1c_, these were shown to not be significant after stricter testing corrections using FDR. Furthermore, LEfSe-analyses failed to report significantly discriminative features at different HbA_1c_ levels.

**Table 3:** Spearman's correlation coefficients between HbA_1c_ and alpha diversity indices in plaque and saliva samples (n = 18).

	Plaque	P-value	Saliva	P-value
**S**_**obs**_	- 0.150	0.552	- 0.264	0.289
**Chao1**	0.067	0.791	- 0.282	0.256
**Shannon**	- 0.237	0.344	- 0.242	0.333
**Simpson**	0.220	0.380	0.195	0.437

S_obs_, total observed species

Six of the 18 participants qualified for a second examination after demonstrating significant improvement of glycemic control after 3 months (Table 4). Five of these six patients had a baseline HbA_1c_ value above the baseline median (HbA_1c_ median = 8.05%). In summary, IGC participants demonstrated a mean HbA_1c_ reduction of 1.57% (minimum = 0.8%, maximum = 3.6%). This IGC study population enabled a longitudinal approach for investigating relationships between microbial communities before and after improved glycemic control.

**Table 4:** Anthropometric and clinical data of patients demonstrating improved glycemic control at baseline and after 3 months (IGC population).

	Visit	IGC 1	IGC 2	IGC 3	IGC 4	IGC 5	IGC 6
Sex		m	m	m	f	f	m
**Age (years)**		62	58	67	55	75	66
**BMI (kg/m**^**2**^**) **	Baseline	34.60	31.41	25.22	46.23	29.05	35.92
	Visit 2	33.56	28.29	24.90	43.97	27.99	35.92
**Waist-hip ratio **	Baseline	0.94	1.04	0.93	0.92	0.90	1.08
	Visit 2	0.95	1.01	0.97	0.93	0.89	1.06
**RR systolic (mmHg)**	Baseline	180	144	122	156	148	153
	Visit 2	152	135	122	145	125	156
**RR diastolic (mmHg)**	Baseline	96	81	85	88	71	77
	Visit 2	87	73	95	89	68	81
**Triglycerides (mM) **	Baseline	1.71	1.46	1.00	2.85	1.37	1.77
	Visit 2	1.16	0.54	0.97	2.23	1.06	1.82
**Total cholesterol (mM) **	Baseline	5.54	5.47	4.57	5.54	4.18	2.90
	Visit 2	5.42	4.39	4.47	4.66	3.32	2.82
**LDL-C (mM) **	Baseline	3.91	3.71	2.94	3.32	2.66	1.41
	Visit 2	4.01	3.03	2.95	2.75	1.93	1.33
**HDL-C (mM) **	Baseline	0.95	1.37	1.35	1.49	1.09	0.98
	Visit 2	0.99	1.47	1.37	1.40	1.07	1.02
**HbA**_**1c**_ **(%) **	Baseline	9.1	7.5	8.2	13.5	8.3	8.4
	Visit 2	7.8	5.9	6.9	9.9	7.5	7.6

HbA_1c_, glycosylated haemoglobin; LDL-C, low-density lipoprotein cholesterol; HDL-C, high-density lipoprotein cholesterol; m, male; f, female, Visit 2: three month after baseline

**Fig 2:**
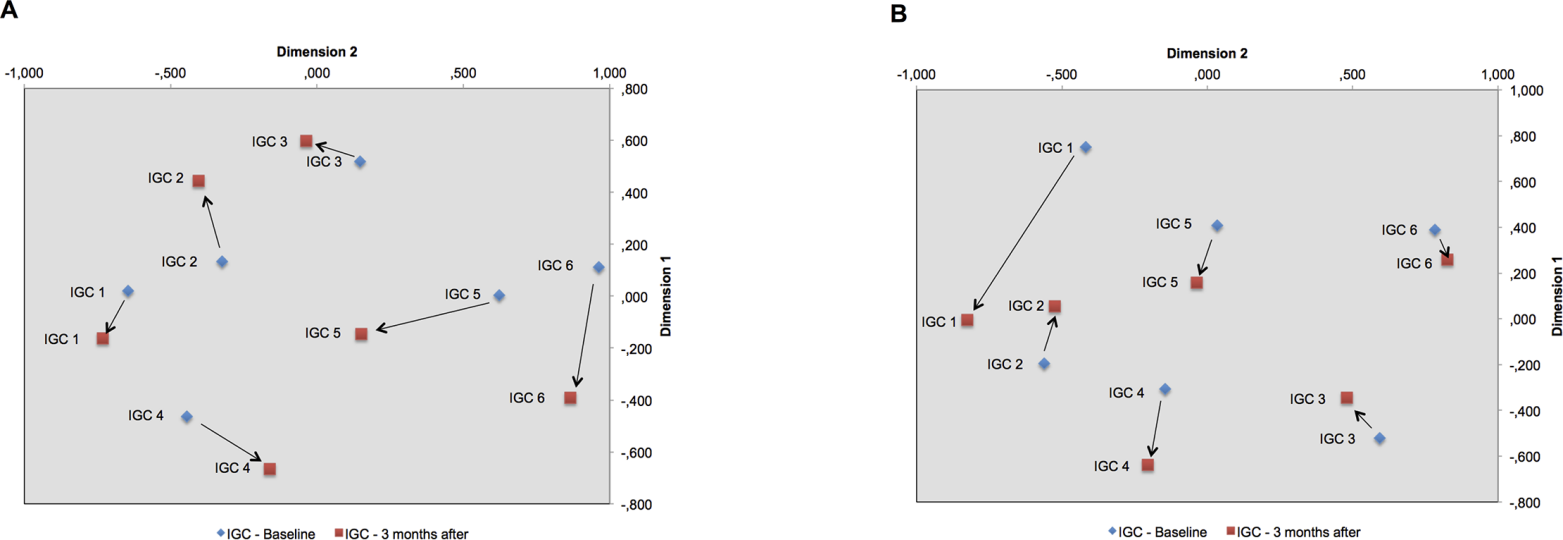
Multi-dimensional scaling based on Bray-Curtis distance in plaque (A) and in saliva (B) before and after improvement of glycemic control.

In both, plaque and saliva samples, all alpha diversity metrics failed to report a difference in richness or diversity between the two examinations (Wilcoxon rank sum p > 0.05) (Table 5). In addition, LEfSe-analysis as well as beta diversity analyses (Bray-Curtis-distance) also reported no differences. Using MDS based on Bray-Curtis-distance, the directional change of community composition following improved glycemic control was inconsistent and no uniform trend could be found (Fig 2).

**Table 5:** Comparison of alpha diversity measures (Mean ± SD) in participants demonstrating improved glycemic control (n = 6).

	Visit	Plaque	P-value^a^	Saliva	P-value^a^
**S**_**obs**_	Baseline	169.00 ± 37.75	0.075	167.67 ± 56.96	0.753
	Visit 2	151.33 ± 57.33		161.33 ± 60.29	
**Chao1**	Baseline	194.76 ± 32.46	0.116	188.09 ± 59.52	0.463
	Visit 2	168.75 ± 56.45		181.92 ± 61.28	
**Shannon**	Baseline	3.17 ± 0.74	0.753	3.27 ± 0.47	0.345
	Visit 2	3.26 ± 0.73		3.01 ± 0.47	
**Simpson**	Baseline	0.13 ± 0.10	0.917	0.08 ± 0.03	0.345
	Visit 2	0.10 ± 0.07		0.11 ± 0.04	

S_obs_, total observed species; Visit 2, three month after baseline;

^a^ Wilcoxon-rank-sum test

### Obesity and the composition of oral microbiome

**Fig 3:**
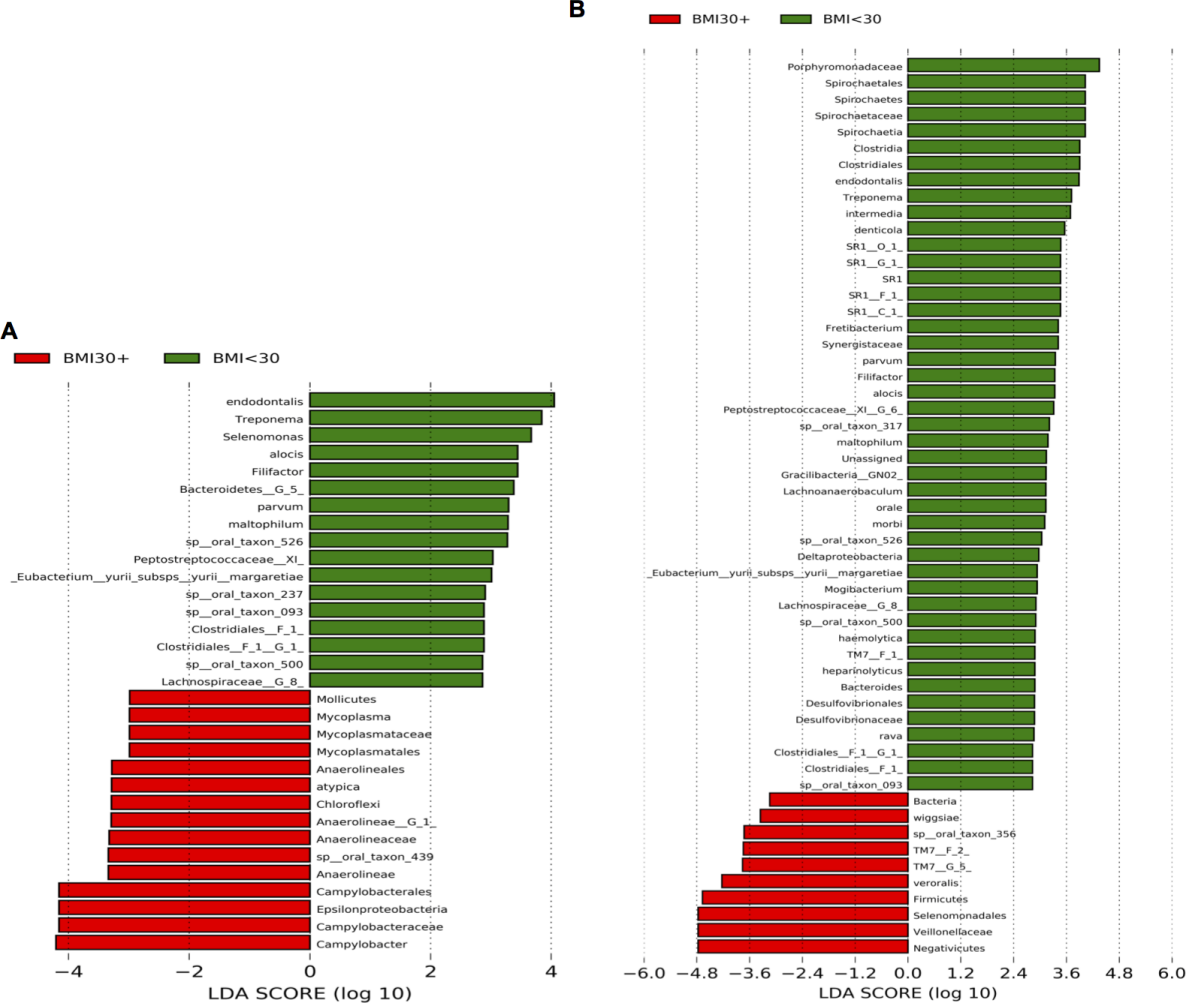
Histogram of the computed LDA scores for features that are differential among conditions of interest with statistical and biological significance, ranked according to the effect size. Conditions compared were (i) BMI < 30 kg/m^2^ (non-obese) and (ii) BMI ≥ 30 kg/m^2^ (obese, BMI +) in plaque (A) and in saliva (B).

In order to analyse the potential association between body weight and the composition of the oral microbiome, the LEfSe analysis was repeated to search for significantly discriminative features between obese (BMI ≥ 30 kg/m^2^, n = 6) and non-obese patients with BMI < 30 kg/m^2^ (controls, n = 12). 32 significantly discriminative features were identified in plaque, and 55 in saliva (Fig 3). Especially in saliva, two interesting observations were made: (i) over four times as many discriminative features were found in the non-obese group, and (ii) the identification of the *Firmicutes* phylum as a significantly discriminative feature at over four orders of magnitude in patients with obesity.

Further, univariate Spearman's correlation analyses reported a statistically significant correlation between BMI and community richness/diversity as measured by alpha diversity metrics (Table 6). This observation was pronounced in plaque. An increase of BMI led to a significant reduction of S_obs_, Chao1 and Shannon indices, and to a significant increase in the Simpson index. Thus, the conclusion can be made that BMI is negatively correlated with species richness and diversity in plaque. In saliva, measures of species richness (S_obs_ and Chao1) were found to be significantly inversely correlated with BMI.

**Table 6:** Spearman's correlation coefficients between BMI and different alpha diversity in plaque and saliva samples.

	Plaque	P-value	Saliva	P-value
**S**_**obs**_	- 0.595	0.009	- 0.580	0.012
**Chao1**	- 0.517	0.028	- 0.604	0.008
**Shannon**	- 0.573	0.013	- 0.461	0.054
**Simpson**	0.519	0.027	0.234	0.349

S_obs_, total observed species

## Discussion

In summary, the oral microbial composition was found to differ significantly between obese and non-obese subjects. Furthermore, a negative correlation between BMI and species diversity was observed in both subgingival plaque and in saliva. However, associations between the oral microbiome and glycemic level could not be replicated using cross-sectional or longitudinal approaches.

A large body of evidence supports the hypothesis that the gut microbiota may be associated with obesity. Interventional studies performed in the human and mouse model using fecal microbiota transplantation supported the causal effect of the gut microbiome on obesity [7–9]. The proposed pathophysiological mechanisms, with which the gut microbiota could contribute to the development of obesity, are not fully understood. Thus far, pathways describing (i) increased energy harvesting, (ii) the induction of inflammatory responses as well as (iii) a variety of metabolic and immune interactions by the gut microbiota have been of major interest. Bacteria specific pathways allow increased caloric uptake through microbial fermentation of otherwise indigestible dietary polysaccharides into absorbable monosaccharides [7,34]. Short chain fatty acids, specifically butyrate, acetate and propionate, are microbial-fermentation products directly involved in hepatic gluconeogenesis and cholesterol synthesis [35], and have also been shown to increase expression of the adipokine leptin [36,37]. Constant exposition to bacterial lipopolysaccharides (LPS) of gram-negative intestinal bacteria triggers a chronic low-grade inflammation, which has been shown to trigger body weight gain and insulin resistance [38]. In severe periodontitis, gram-negative anaerobes tend to dominate in the periodontal pocket [39]. The ulceration of the epithelium lining of periodontal pockets resulting from periodontitis represents a direct entry point for periodontal pathogens and bacterial products (e.g. LPS) into the systemic circulation. Thus, the oral microbiota could, together with intestinal bacteria, play a role in systemic inflammation and be involved in the microbiota-obesity-axis. Indeed, accumulating epidemiologic evidence has revealed a significant association between periodontitis and obesity [12,40].

Data from our study of the microbial composition within the oral cavity yielded some novel associations and also confirmed some previous findings. Our LEfSe analyses revealed distinct significantly discriminative features between two conditions (obese and non-obese patients). In subgingival plaque, LEfSe revealed a dominance of *Bacteroidetes*, *Spirochaetes* and *Firmicutes* in the population without obesity. In obese patients, *Proteobacteria*, *Chloroflexi* and *Firmicutes* were overrepresented, with the noted absence of representatives from the phylum *Bacteroidetes*. LEfSe analysis revealed a similar observation in saliva: in the control population, *Bacteroidetes* and *Firmicutes*, among others, were overly abundant. Notably, the phylum *Firmicutes* was identified in obese patients as an independent significantly discriminative feature with an abundance of over four orders of magnitude. These findings in the oral cavity mainly correspond to the hypothesis that the obesity-associated microbiota of the gut is characterized by reduced abundance of *Bacteroidetes* paralleled by an increased abundance of *Firmicutes* resulting in in lower ratios of *Bacteroidetes* to *Firmicutes* [3,34]. Importantly, the model that the *Bacteroidetes/Firmicutes* ratio alone may be linked to obesity might in fact be incomplete, as other factors may also be involved in the aetiology of this multifactorial metabolic disease. The first reported gut metagenomic analysis characterizing type 2 diabetes mellitus patients with obesity before and three months after bariatric surgery showed dramatic changes in the individual composition of the gut microbiota [4]. In particular, a substantial shift at the phylum level towards *Proteobacteria* with simultaneous decreases of both *Firmicutes* and *Bacteroidetes* after surgery was found. This shift in the microbial ecology of the gut was accompanied by marked reduction of BMI, significant improvement of the metabolic state and reduced inflammatory activity.

Beside different composition of oral microbiota in obese and non-obese subjects, reduced species diversity in the oral cavity of obese patients was pronounced in our study population. This result is in line with similar observations demonstrating reduced microbial diversity identified in the distal gut [3,5] as well as in the upper gastrointestinal tract [6] and which has been linked with obesity.

Although our results were limited to obesity within a cohort of patients with type 2 diabetes mellitus, it is plausible that obesity would also be associated with the oral microbiota in non-diabetic subjects. Studies mentioned above linking the microbiota of the gut and the upper digestive tract under non-diabetic conditions supports that hypothesis. In addition, the potential involvement of oral bacteria in obesity has also been investigated in healthy non-diabetes subjects. In saliva, Goodson et al. identified *Selenomonas noxia*, a representative of the *Firmicutes* phylum, to be a robust predictor of obesity [40]. The microbiotas of the oral cavity and the upper gastrointestinal tract are very similar [16], and oral pathogens may contribute to dysbiosis in the gut microbiota leading to impaired barrier function and systemic inflammation [17,18]. Obesity itself, is increasingly being associated with higher proportions of different periodontal pathogens [41,42].

Given the established inter-relationships between type 2 diabetes and periodontitis, it was anticipated and indeed observed that the study population of patients with type 2 diabetes mellitus presented with a high prevalence of periodontitis. The majority of participants (94.4%) were diagnosed with moderate or severe forms of periodontitis. An important consideration at this point is the inclusion criterion for the study: participants were required to have at least ten teeth (wisdom teeth excluded). The exclusion of edentulous patients or patients not meeting the inclusion criterion could have skewed the periodontitis prevalence. Given that the study population did not include an age-matched healthy population, comparisons with similar studies which included healthy, prediabetic and diabetic populations show that the results of the given study are likely in keeping with this consensus [43].

The effect of diabetes on the oral microbiome and its role in the aggravation of periodontitis remains unclear. There exist numerous studies demonstrating contrary results regarding a possible association between altered glucose metabolism and changes in the periodontal microbiome [14,44]. Currently, there is no consistent evidence of causal relation between glycemic state and periodontal microbial dysbiosis [44]. We were unable to replicate any associations between the oral microbiome and glycemic level. In the studies demonstrating a microbial shift as a result of diabetes [45–47], it is important to note that none of the three studies adjusted for body weight as a potential confounding factor. This may explain the contrary results as our study also demonstrates that body weight is associated with changes in the composition and diversity of the oral microbiota.

While our findings regarding obesity were largely in line with previous studies, this methodology presented with possible limitations that may have obscured the underlying relationship. The first limitation was the lack of a lean control group. In this case, a healthy weight group (18.5–25.0 kg/m^2^) was not included due to the study design. Our two groups only had a resolving power to differentiate between obese patients (BMI ≥ 30 kg/m^2^) and patients who were still overweight (BMI < 30 kg/m^2^). Secondly, the low resolving power as a result of the small sample size represents another potential limitation. It is well established that a reduction of HbA_1c_ and blood pressure significantly reduces microvascular complications in patients with diabetes. A cross-sectional study from NHANES between 1988 and 2010 showed that while there appears to be a dramatic increase in patients with diabetes meeting the goals set by the ADA, there is still much room for improvement [48]. The authors also concluded that achieving the ADA recommendations may be biologically unattainable for some patients due to disease severity and other comorbidities. Glycemic control is also complicated in patients presenting with more severe ß-cell loss. Other factors including lack of management skills or lack of adherence to demanding self-care regiments, and an aversion to lifestyle changes are often attributed to complications of diabetes management. Given this, it was anticipated that only 4 of the 18 examined patients reached the ADA recommendation of HbA_1c_ < 7.0%, despite many years of treatment with clinicians and diabetes educators. In the case of our study, only 6 participants demonstrated an improvement in glycemic control of at least of 0.5% HbA_1c_ intensified diabetes treatment during the entire observation period of 3 months.

The oral cavity could represent a relevant surrogate representation of the gut microbiome [49]. Our study gives weight to previous findings that alterations in the oral microbiome have potential in sentinel diagnostic and prognostic application [49–51]. In conclusion, the results of the here presented study provide clues that oral bacteria may be involved in pathways leading to obesity, and this promising aspect warrants future examinations.

## Supporting information

S1 Table Absolute abundances of identified OTUs. (XLSX)
